# Activation and Immune Regulation Mechanisms of PYHIN Family During Microbial Infection

**DOI:** 10.3389/fmicb.2021.809412

**Published:** 2022-01-25

**Authors:** Xiaojiao Fan, Lianying Jiao, Tengchuan Jin

**Affiliations:** ^1^Department of Obstetrics and Gynecology, The First Affiliated Hospital of USTC, Division of Life Sciences and Medicine, University of Science and Technology of China, Hefei, China; ^2^Department of Biochemistry and Molecular Biology, School of Basic Medical Sciences, Xi’an Jiaotong University Health Science Center, Xi’an, China; ^3^Institute of Molecular and Translational Medicine, Translational Medicine Institute, Xi’an Jiaotong University Health Science Center, Xi’an, China; ^4^The CAS Key Laboratory of Innate Immunity and Chronic Disease, School of Basic Medical Sciences, Division of Life Sciences and Medicine, University of Science and Technology of China, Hefei, China; ^5^CAS Center for Excellence in Molecular Cell Science, Shanghai, China

**Keywords:** innate immunity, PRR, PAMP, PYHIN family, AIM2, IFI16, p202, p204

## Abstract

The innate immune system defenses against pathogen infections via patten-recognition receptors (PRRs). PRRs initiate immune responses by recognizing pathogen-associated molecular patterns (PAMPs), including peptidoglycan, lipopolysaccharide, and nucleic acids. Several nucleic acid sensors or families have been identified, such as RIG-I-like receptors (RLRs), Toll-like receptors (TLRs), cyclic GMP-AMP synthase (cGAS), and PYHIN family receptors. In recent years, the PYHIN family cytosolic DNA receptors have increased attention because of their important roles in initiating innate immune responses. The family members in humans include Absent in melanoma 2 (AIM2), IFN-γ inducible protein 16 (IFI16), interferon-inducible protein X (IFIX), and myeloid cell nuclear differentiation antigen (MNDA). The PYHIN family members are also identified in mice, including AIM2, p202, p203, p204, and p205. Herein, we summarize recent advances in understanding the activation and immune regulation mechanisms of the PYHIN family during microbial infection. Furthermore, structural characterizations of AIM2, IFI16, p202, and p204 provide more accurate insights into the signaling mechanisms of PYHIN family receptors. Overall, the molecular details will facilitate the development of reagents to defense against viral infections.

## Introduction

The innate immune system can utilize pattern recognition receptors (PRRs) to detect and defense against invading pathogens. Once PRRs recognize pathogen-associated molecular patterns (PAMPs), peptidoglycan, lipopolysaccharide, and nucleic acids induce innate immune responses. A series of PRRs capable of sensing nucleic acids from pathogens have been identified over the last decades. Retinoic acid-inducible gene I (RIG-I)-like receptors (RLRs), including RIG-I, melanoma differentiation-associated protein 5 (MDA5), and laboratory of genetics and physiology 2 (LGP2), are responsible for detecting pathogen-derived RNA in the cytosol (Berke et al., 2013). Endosomal Toll-like receptors (TLRs) include TLR3, TLR7, and TLR8 sense RNA, while TLR9 is a DNA sensor in the endosome (Hemmi et al., 2000; Alexopoulou et al., 2001; Bauer et al., 2001; Ahmad-Nejad et al., 2002). In addition, cyclic GMP-AMP synthase (cGAS) senses cytosolic double-strand DNA (dsDNA) (Sun et al., 2013; Wu et al., 2013). Human PYHIN family receptors Absence in melanoma 2 (AIM2) (Hornung et al., 2009), IFN-γ inducible protein 16 (IFI16), Interferon-inducible protein X (IFIX, or pyrin and HIN domain family member 1, PYHIN1) (Diner et al., 2015), and mouse p204 (Unterholzner et al., 2010; Horan et al., 2013; Zhu et al., 2014) sense DNA in the cytosol. Furthermore, IFI16, p204, and IFIX also sense DNA in the nucleus. Lastly, other cytosolic DNA sensors have been reported, including DNA dependent activator of IFN regulatory factor (DAI, also called Z-DNA-binding protein 1, ZBP1) (Takaoka et al., 2007; Jiao et al., 2020), RNA polymerase III, leucine-rich repeat-containing protein (LneRRF1P1) (Yang et al., 2010), Ku70/Ku80 protein (Zhang X. et al., 2011), DEAH box polypeptide 9 (DHX9) and DEAH box polypeptide 36 (DHX36) (Kim T. et al., 2010), and DDX41 helicase (Zhang Z. et al., 2011).

**FIGURE 1:**
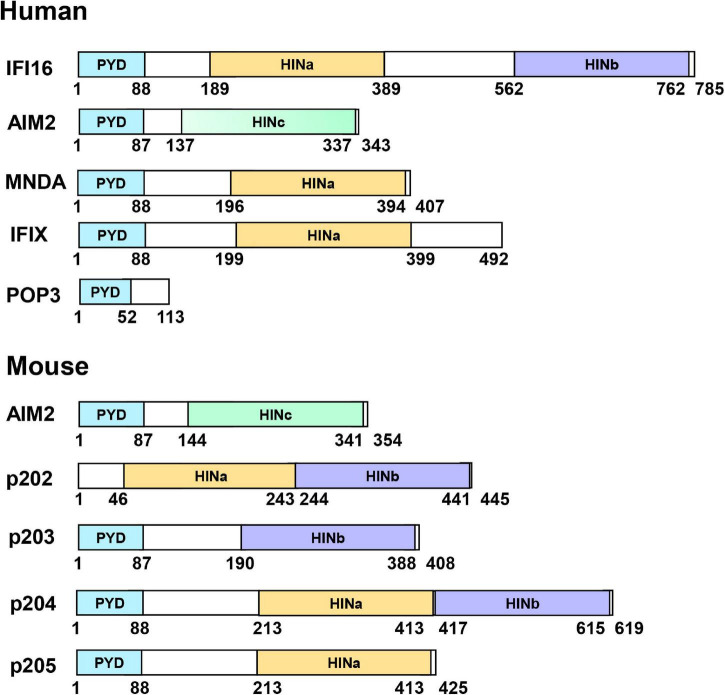
Domain organization of PYHIN family proteins. PYHIN family members possess an N-terminal pyrin domain (PYD) and one or two C-terminal HIN domains, classified as three subtypes; HIN A, HIN B, or HIN C. p202 from mouse lacks PYD and POP3 from human lacks of HIN domains.

PYHIN family (also called p200 protein) are DNA sensors activated by pathogens infection and stress conditions such as DNA break. In humans, these family members include AIM2, IFI16, myeloid cell nuclear differentiation antigen (MNDA) (Briggs et al., 1992), IFIX. Sometimes the pyrin domain only protein 3 (POP3) was also considered as a variant of the PYHIN family, which is resulted from a HIN domain deletion (Khare et al., 2014), while members in mouse include AIM2, p202, p203, p204, and p205 (Ludlow et al., 2008). Most PYHIN family members possess an N-terminal pyrin domain (PYD, or PAAD or DAPIN domain) and one or two C-terminal hematopoietic interferon-inducible nuclear antigens with 200 amino acid repeats (HIN or HIN-200) domain (Figure 1). p202 lacks a PYD and only harbors two HIN domains. POP3 only possesses a PYD and lacks a HIN domain. PYD belongs to the death domain (DD) superfamily and forms interactions with other PYD-containing proteins to form higher complexes (Park et al., 2007; Jin and Xiao, 2015). PYD: PYD interactions regulate various cellular processes, ranging from inflammation and immunity to apoptosis and cell cycle (Stehlik, 2007). HIN domains have been classified into three subtypes: A, B, and C. MNDA and IFIX contain a single type A HIN (HINa) domain, whereas IFI16, p202, and p204 have one HINa and one HINb domain. p203 has a single HINb domain, whereas AIM2 has a single type C HIN (HINc) domain. HIN domains are responsible for binding DNA. In addition to DNA sensing and subsequence immune activation, it was reported that PYHIN family members function in cell growth and cell cycle control (Lembo et al., 1998; Dauffy et al., 2006), apoptosis (Aglipay et al., 2003), senescence (Xin et al., 2004), DNA damage response, tumor suppression (Chen et al., 2006), and differentiation and autoimmunity (Dermott et al., 2004).

In this article, we briefly review the recent advances in understanding the activation and immune regulation mechanisms of the PYHIN family during microbial infection, especially in defending against viral infection. Structural insights into AIM2, IFI16, p202, and p204 provide more accurate DNA recognition mechanisms and signaling transduction pathways. Furthermore, the molecular details will facilitate the development of reagents to defense against pathogen infections.

## Absent in Melanoma 2

Absent in melanoma 2 (AIM2) is a 39 kDa protein expressed in the spleen, small intestine, and peripheral leukocytes (DeYoung et al., 1997). It is associated with psoriasis, systemic lupus erythematosus (SLE), chronic kidney disease, diabetes, atherosclerosis, and neuronal diseases (Kimkong et al., 2009; Dombrowski et al., 2011; Yang et al., 2015; Sharma et al., 2019). As a tumor suppressor, the dysregulation of AIM2 is associated with colon and small bowel cancers (Schulmann et al., 2005; Woerner et al., 2007), hepatocellular carcinoma, and prostate cancer (Ponomareva et al., 2013; Ma et al., 2016; Chen et al., 2017), Epstein-Barr virus (EBV)-associated nasopharyngeal carcinoma (Chen et al., 2012), human cutaneous squamous carcinoma (Farshchian et al., 2017), human papillomavirus (HPV)-associated cervical cancer (Milutin Gasperov et al., 2014), non-small cell lung cancer (Kong et al., 2015). Hu et al. (2016) found AIM2 could detect irradiation-induced DNA damage and assemble into inflammasome in the nucleus. AIM2 was later identified as a cytosolic dsDNA sensor that can assemble into inflammasome with ASC (apoptosis-associated speck-like protein containing a CARD) and pro-caspase-1 (Burckstummer et al., 2009; Fernandes-Alnemri et al., 2009; Hornung et al., 2009; Roberts et al., 2009). AIM2 inflammasome can be activated by bacterial pathogens such as *Francisella tularensis* (Jones et al., 2010), *Listeria monocytogenes* (Kim S. et al., 2010; Rathinam et al., 2010; Sauer et al., 2010; Warren et al., 2010; Wu et al., 2010; Ge et al., 2012), *Streptococcus pneumoniae* (Fang et al., 2011), *Mycobacterium tuberculosis* (Saiga et al., 2012), *Staphylococcus aureus* (Hanamsagar et al., 2014), and *Aspergillus fumigatus* (Karki et al., 2015). The activation of AIM2 inflammasome relies on type I interferons during *Francisella novicida* infection (Belhocine and Monack, 2012; Man et al., 2015). Recently, it was reported that AIM2 could enhance the stability of T regulatory cells (Tregs) during inflammation (Chou et al., 2021). Later, Fidler et al. (2021) found activated AIM2 inflammasome in *Jak2^V617F^* macrophages could aggravate atherosclerosis. Lee et al. (2021) reported that AIM2, pyrin, and ZBP1 could form a PANoptosome complex to drive PANoptosis for host defense during HSV-1 and *Francisella novicida* infections.

AIM2 also senses dsDNA from virus such as HPV 16 (Reinholz et al., 2013), hepatitis B virus (HBV) (Zhen et al., 2014), and EBV (Torii et al., 2017). Human herpesviruses herpes simplex type 1 (HSV-1) and Kaposi’s sarcoma-associated herpesvirus (KSHV) activate the inflammasome in an AIM2-independent manner. Mouse cytomegalovirus (MCMV) and vaccinia virus (VACV) infection induce caspase-1 activation and IL-1β secretion in an AIM2-dependent manner (Hornung et al., 2009; Rathinam et al., 2010). Additionally, several RNA viruses such as Chikungunya virus (CHKV) or West Nile virus (Ekchariyawat et al., 2015) and enterovirus A71 can also activate AIM2 inflammasome, but the mechanism remains unknown (Yogarajah et al., 2017).

### Molecular Mechanism of Absent in Melanoma 2 Inflammasome Assembly

**FIGURE 2:**
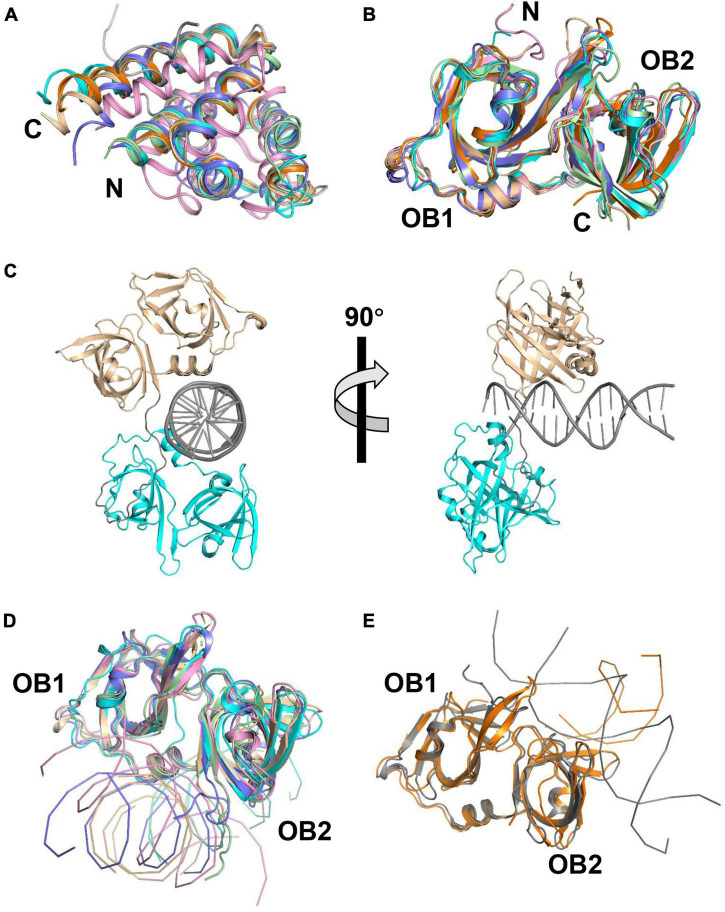
Structures of PYHIN family proteins. **(A)** Structural superposition of PYD of AIM2, MNDA, and p205. The structure of human AIM2 PYD with MBP (PDB: 3VD8) and its mutant (F27G, PDB: 4O7Q) are shown in cyan and pale green. The structure of mouse AIM2 PYD (PDB: 2N00) is shown in slate. Structures of human MNDA PYD without MBP (PDB: 5WPZ) and with MBP (PDB: 5H7Q, 5WQ6) are shown in pink, orange, and wheat. The solution structure of p205 PYD (PDB: 2YU0) is shown in gray. **(B)** Structural superposition of HIN domains of IFI16, p202, and p204. Structures of p204 HINa (PDB: 5YZP) and HINb (PDB: 5YZW) are shown in wheat and cyan. Structures of IFI16 HINa (PDB: 2OQ0) and HINb (PDB: 3B6Y) are shown in orange and slate. Structures of p202 HINa (PDB: 4JBJ) and HINb (PDB: 4L5T) are shown in pink and pale green. **(C)** Crystal structure of p204 HINab: dsDNA complex. Structures of p204 HINa and HINb are shown in wheat and cyan. dsDNA and the linker between HINa and HINb are shown in gray. **(D)** Similar DNA-binding mode of AIM2, IFI16 HINb, and p204 HINa and HINb. Structures of p204 HINa: DNA (wheat) and HINb: DNA (cyan) are from HINab: dsDNA complex (PDB: 5Z7D). Structures of human AIM2 HIN (PDB: 3RN2), mouse AIM2 HIN (PDB: 4JBM), and IFI16 HINb (PDB: 3RNU) are shown in pink, pale green, and slate, respectively. **(E)** Similar DNA-binding mode of IFI16 HINa and p202 HINa. Structures of p202 HINa (PDB: 4L5R) and IFI16 HINa (PDB: 4QGU) are shown in gray and orange, respectively.

Three structures of AIM2 PYD (AIM2^PYD^) have been reported, including mouse AIM2^PYD^ (mAIM2^PYD^) (Hou and Niu, 2015), wild-type human AIM2^PYD^ (hAIM2^PYD^), and its F27G mutant (hAIM2-F27G^PYD^) (Jin et al., 2013; Lu et al., 2014a). AIM2^PYD^ exhibits a globular structure of a six-helix bundle, sharing the common feature of typical death domains (Figure 2A). Except for the minor differences around α2-α3 helix, the structure of AIM2^PYD^ in the cryo-electron microscopic (Cryo-EM) is similar to its crystal structure, indicating the structural plasticity of AIM2^PYD^ is vital in the PYD: PYD interaction (Lu et al., 2015). Lu et al. (2014b,2015) reported the cryo-EM structure of GFP-hAIM2^PYD^ and human PYD of ASC helical filaments. Electrostatic and hydrophobic interactions are necessary for AIM2^PYD^ polymerization confirmed by EM analysis, pull down, and yeast two-hybrid assay (Lu et al., 2014a; Morrone et al., 2015; Hafner-Bratkovic et al., 2018). It is proposed that the AIM2^PYD^ filament serves as a platform to nucleate ASC^PYD^ filaments (Lu et al., 2014b). Crystal structure of AIM2 HIN domain (AIM2^HIN^) in complex with dsDNA was determined, and the structure reveals that the HIN domain binds to both the major and minor grooves of dsDNA (Jin et al., 2012). Both oligonucleotide/oligosaccharide (OB) folds and the connecting linker of AIM2^HIN^ participate in dsDNA binding (Figure 2B). It has been reported that a minimum size of ∼ 80 bp dsDNA is required for activating AIM2 and inducing IL-1β production (Jin et al., 2012).

Absent in melanoma 2 presents in an auto-inhibited state in the absence of DNA, confirmed by *in vitro* pull-down assay, ITC, and fluorescence polarization inhibition assays (Jin et al., 2012, 2013). Lu et al. (2015) proposed the AIM2^HIN^: dsDNA filament model, in which AIM2^HIN^ is wrapped around dsDNA filament and the filament diameter is ∼7.5 nm. Each HIN domain interacts with six adjacent HIN domains, contributing to adjacent PYDs and forming short helical filaments. Subsequently, AIM2^PYD^ filaments interact with ASC^PYD^ filaments, and the CARD also organizes into ASC^CARD^ filaments to nucleate caspase-1 filaments. Lastly, caspase-1-activating inflammasome induces the production of mature IL-1β and IL-18.

### Regulation of Absent in Melanoma 2

AIM2 inflammasome is activated by the elevated expression of Histone deacetylases 3 (HDAC3) and downregulated by RGFP966 (an HDAC3 inhibitor) (Zhang et al., 2020). Tripartite motif protein 11 (TRIM11) negatively regulates AIM2 by mediating the degradation of AIM2 (Liu et al., 2016; Yang et al., 2017). IFI16 isoform IFI16-β inhibits AIM2 via the competition for dsDNA and interaction with AIM2 (Wang et al., 2018). p202 and POP3 also inhibit AIM2 activation (Roberts et al., 2009; Khare et al., 2014). Additionally, the virus also inhibits AIM2 inflammasome as a defense strategy. Herpes simplex virus 1 (HSV-1) tegument protein VP22 and Human cytomegalovirus (HCMV) protein pUL83 (also named pp65) interact with AIM2 to inhibit the oligomerization and activation of AIM2 (Huang et al., 2017; Maruzuru et al., 2018).

### Signal Transduction Pathway of Absent in Melanoma 2

**FIGURE 3:**
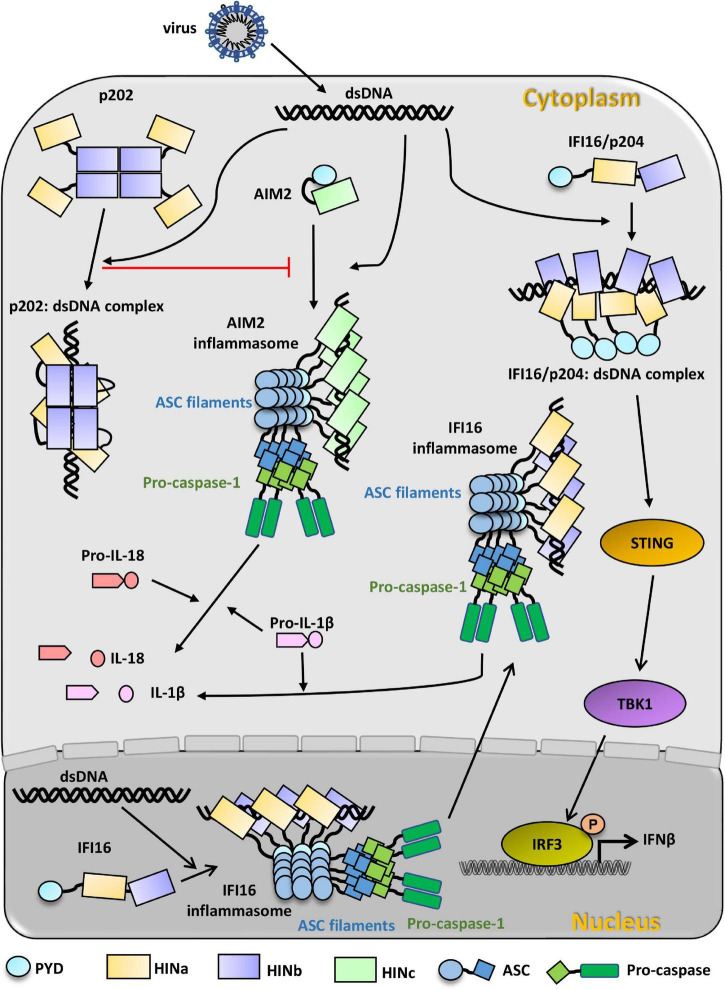
PYHIN family proteins in innate immunity recognition of viral dsDNA. Cytosolic dsDNA from invading virus activates AIM2, which presents an auto-inhibition state before recognizing viral dsDNA. AIM2 HIN domain binds to dsDNA in the cytosol and subsequently binds to the adapter ASC through PYD: PYD interaction. AIM2 inflammasome is formed through AIM2-ASC-procaspase-1 oligomerization. Activated caspase-1 can directly cleave pro-IL-1β and pro-IL-18 to IL-1β and IL-18, which can respond to infection. p202 exists as a tetramer in the cytosol and can also bind to dsDNA from invading viruses. p202 can inhibit the formation of AIM2 inflammasome. IFI16 presents an extended state before recognizing dsDNA. In the nucleus, viral dsDNA binds to IFI16 HIN domains and induces IFI16 oligomerization with the assistant of ASC and pro-caspase-1, and the oligomer migrates to the cytoplasm to cleave pro-IL-1β to IL-1β. In the cytosol, viral dsDNA can also directly initiate the activation of IFI16/p204 independent of the inflammasome. Multiple IFI16/p204 HINab domains synergistically bind to the long dsDNA, resulting in the adjacent PYD aggregate to activate STING and TBK1. Activated TBK1 phosphorylates IRF3 and induces the production of IFNβ to defend against the virus infection.

AIM2 is in an auto-inhibited state in the cytoplasm (Figure 3). Once AIM2 senses the invasion of viral dsDNA, its HIN domain interacts with dsDNA. Then AIM2 associates with the adaptor protein ASC through PYD: PYD interaction. ASC further recruits and activates pro-caspase-1 through a homotypic CARD: CARD interaction. Caspase-1, in turn, processes the inactive precursors of IL-1β and IL-18 into mature cytokines. The activation of caspase-1 results in a rapid inflammatory form of cell death. dsDNA also acts as a platform for recruiting multiple AIM2 molecules, promoting the close approximation of AIM2^PYD^ and the formation of AIM2 inflammasome. AIM2 inflammasome, in turn, facilitates the nucleation of ASC^PYD,^ leading to the formation of ASC filaments and activation of caspase-1. However, the signal transduction pathway of AIM2 can be inhibited by p202, IFI16 isoform IFI16-β, and POP3.

## IFN-γ Inducible Protein 16

IFN-γ inducible protein 16 (IFI16) is an 82 kDa protein and contains two HIN domains (HINa and HINb) (Figure 1). It is engaged in cell cycle control and transcriptional regulation. It can interact with transcription factors Sp1 and p53 (Johnstone et al., 2000; Liao et al., 2011). It can also bind to the promoter region of oncogenes like c-MYC and RAS to repress their transcription (Zhang et al., 2007; Egistelli et al., 2009). By cooperating with p53, IFI16 can inhibit tumorigenesis (Fujiuchi et al., 2004; Choubey and Panchanathan, 2016; Lin et al., 2017). It is also associated with the retinoblastoma tumor-suppressor protein Rb and the transcription factor E2F1 to mediate transcriptional repression (Xin et al., 2003). It can interact with Sp1 to inhibit viral replication (Gariano et al., 2012). The aberrant activity of IFI16 is also associated with several autoimmune disorders, such as SLE and Sjögren syndrome (Seelig et al., 1994; Uchida et al., 2005; Mondini et al., 2007, 2010; Kimkong et al., 2009; Veeranki and Choubey, 2012; Gugliesi et al., 2013; Alunno et al., 2015; Baer et al., 2016). IFI16 was initially identified as a novel DNA sensor by affinity pull-down using cytosolic extracts from human THP-1 monocytes (Unterholzner et al., 2010). It can form inflammasomes sensing DNA viruses replicating in the nucleus (Kerur et al., 2011). It also can response to infection with retroviruses such as human immunodeficiency virus type 1 (HIV-1) in macrophages (Jakobsen et al., 2013) as well as to infection with intracellular bacteria such as *Listeria monocytogenes* (Hansen et al., 2014) and *Francisella novicida* (Storek et al., 2015).

IFI16 senses dsDNA from several viruses, including KSHV and VACV (Unterholzner et al., 2010; Kerur et al., 2011; Ansari et al., 2015), HSV-1 (Johnson et al., 2013), EBV (Ansari et al., 2013), and HIV-1 (Jakobsen et al., 2013). It also recognizes ssDNA from HIV-infected CD4^+^ T cells and nuclear-damaged DNA from etoposide-treated keratinocytes (Monroe et al., 2014; Dunphy et al., 2018). Yang et al. (2020) reported that IFI16 could recognize the covalently closed circular DNA (cccDNA) of HBV and mediate the epigenetic silencing of HBV gene expression (Yang et al., 2020). IFI16 can also reduce viral replication in cells carrying the episomal HPV 18 genome (Lo Cigno et al., 2015) and reduce HCMV mRNAs synthesis (Griffiths et al., 2015). Wichit et al. (2019) found that IFI16 could inhibit the replication of Zika (ZIKV) and CHKV in primary foreskin fibroblasts (Wichit et al., 2019). Additionally, IFI16 was found to participate in the innate immunity via interacting with RIG-I (retinoic acid-inducible gene I) with its PYD, which leads to the activation of RIG-I (Sui et al., 2014; Jiang et al., 2021). Moreover, IFI16-dependent inflammasome was activated by sensing herpes viruses such as KSHV, HSV-1, and EBV (Kerur et al., 2011; Ansari et al., 2013; Johnson et al., 2013). HSV-1 and HCMV infection initialed the IFI16 dependent IFN-β induction via the STING/TBK1 pathway (Unterholzner et al., 2010; Diner et al., 2016).

### Structural Basis of IFN-γ Inducible Protein 16 Sensing Viral DNA

It has been reported that HINa and HINb domains of IFI16 bind to both ssDNA and dsDNA *in vitro*, and the HINb domain possesses a stronger DNA binding affinity (Yan et al., 2008; Unterholzner et al., 2010; Jin et al., 2012). Structural superimposition reveals that the overall topologies of IFI16 HINa (PDB: 2OQ0) and HINb (PDB: 3B6Y) are similar (Figure 2B). Comparing the structures of IFI16 HINa in HINa: DNA complex (PDB: 4QGU) and HINb in HINb: DNA complex (PDB: 3RNU), HINa and HINb bind to DNA at different surfaces (Figures 2D,E), IFI16 HINa binds DNA via the loops from OB folds to tether DNA whereas HINb binds DNA via the linker between OB1 and OB2 folds. Ni et al. (2016) reported that the mutants with impaired DNA-binding ability, IFI16 HINa enhanced, but HINb reduced the production of IFN-β, suggesting IFI16 HINa and HINb might play distinct roles during sensing DNA.

The full-length and HINab domain of IFI16 exists as a monomer in solution confirmed by small-angle X-ray scattering (SAXS) analysis (Liao et al., 2011; Ni et al., 2016). IFI16 HINab forms a large oligomer in the presence of 42 mer dsDNA (Ni et al., 2016). Morrone et al. (2014) found IFI16 binds to dsDNA in a length-dependent manner and assembles into filaments on the long dsDNA. They proposed that HIN domains of IFI16 independently bind to dsDNA like beads on a string, which further induces the conditional proximity of PYD to driven cooperative filament formation of IFI16 upon encountering dsDNA (Morrone et al., 2014).

### Regulation of IFN-γ Inducible Protein 16 Pathways

IFI16 possesses an evolutionarily conserved multipartite nuclear localization signal (NLS) for sensing viral DNA in the nucleus (Li et al., 2012). IFI16 can be phosphorylated at unknown sites (Choubey and Lengyel, 1992; Johnstone et al., 1998). Li et al. (2012) identified the phosphorylation sites of IFI16 NLS and indicated that S95, S106, and S153 have little impact on localization. Acetyltransferase p300 and histone deacetylase HDAC can regulate the acetylation status of IFI16 (Li et al., 2012). Li et al. (2012) reported that the overexpression of p300 or the inhibition of HDAC could trigger the cytoplasmic accumulation of IFI16. They also reported that STING negatively regulates IFI16 stability by recruiting the E3 ligase TRIM21 to eliminate the high risk of IFI16 accumulation (Li et al., 2019).

Viruses have evolved several strategies to escape host innate immune responses mediated by IFI16. HBV could negatively regulate the expression of IFI16 in hepatocytes (Yang et al., 2020). HCMV virion protein pUL83 can interact with IFI16 to enhance the transcriptional activity of viral immediate-early promoters (Cristea et al., 2010). Li et al. (2013) found that pUL83 can interact with the PYD of IFI16, resulting in the blocking of antiviral response. pUL38 can also cooperate with pUL97 (HCMV encoded serine/threonine-specific kinase) to phosphorylate IFI16 (Dell’Oste et al., 2014). US28, an HCMV encoded G-protein-coupled receptor, is associated with the degradation of IFI16 in the viral latency (Elder et al., 2019). ICP0, a viral ubiquitin ligase from HSV-1, can induce IFI16 degradation (Orzalli et al., 2012). However, another research reported that ICP0 is neither sufficient nor necessary to degrade IFI16 during HSV-1 infection (Cuchet-Lourenco et al., 2013).

### Signal Transduction Pathway of IFN-γ Inducible Protein 16

IFI16 exhibits an extended state in the absence of invasion DNA (Figure 3). In the cytoplasm, IFI16 binds to dsDNA via its C-terminal HINa and HINb domains. Along the long dsDNA, IFI16 forms filaments with the association of PYD. Subsequently, IFI16 filaments recruit STING and TBK1 to phosphorylate STING. The activated STING induces the phosphorylation of IRF3, leading to IFN-I production (Unterholzner et al., 2010; Orzalli et al., 2012; Jakobsen et al., 2013; Liu et al., 2015; Almine et al., 2017). In the nucleus, the binding of exogenous DNA by IFI16 leads to the formation of IFI16-ASC-procaspase-1 inflammasome and cytoplasmic translocation, resulting in the secretion of proinflammatory cytokine IL-1β to defend against viral infection (Kerur et al., 2011; Ansari et al., 2013; Singh et al., 2013).

### p202

Mouse p202 was discovered as an interferon-inducible protein in 1989 (Kingsmore et al., 1989). Though there are three homologous p202 genes (p202a, b, and c), only p202a and p202b are expressed (Wang et al., 1999). p202 possesses HINa and HINb domain and lacks PYD (Figure 1). It can negatively regulate transcription factors including MYOD1, myogenin, AP-1, and NF-κB (Min et al., 1996; Datta et al., 1998; Ma et al., 2003). In prostate cancer cells, p202 can suppress apoptosis by deregulated expression of E2F1 (Yan et al., 2003). In sensitizing breast cancer cells, p202 exhibits pro-apoptotic effects by binding to NF-κB (Wen et al., 2000). p202 has been reported to regulate cell cycle progression. It may negatively regulate p53 transcriptional activity by binding 53BP1 (Datta et al., 1996). It can inhibit cell growth by modulating p21 and inhibit cell proliferation by interacting with the retinoblastoma tumor suppressor protein (pRb) and the transcription factor E2F (Choubey et al., 1996; Choubey and Gutterman, 1997; Gutterman and Choubey, 1999). Rozzo et al. (2001) found that p202 is a potential susceptibility candidate gene in autoimmune disease SLE. They also found that p202 can negatively regulate AIM2 inflammasome by binding to DNA alongside AIM2 (Roberts et al., 2009).

### Structural Basis of Viral Double-Strand DNA Sensing by p202

Several structures of p202 with and without dsDNA have been reported, revealing the mechanism of p202 sensing viral dsDNA. Crystal structures of p202 HINa (PDB: 4JBJ) and HINb (PDB: 4L5T) are highly similar to the structures of known HIN domains of the PYHIN family (Figure 2B). Four crystal structures of HINa in complex with dsDNA have been reported, including with 20 mer dsDNA (PDB: 4L5R and 4LNQ), 12 mer dsDNA (PDB: 4L5S), and 14 mer dsDNA (PDB: 4JBK) complex structures. Comparing p202 HINa and DNA-bound structures reveals that HINa does not undergo obvious conformational changes upon DNA binding. p202 binds to dsDNA via the loops from both OB1 and OB2 folds, different from AIM2 HIN and IFI16 HINb (Figures 2D,E).

Though p202 HINb alone lacks DNA binding activity, it enhances dsDNA binding for the full-length p202 (Roberts et al., 2009). p202 HINb exists as a tetramer in the solution and presents as dimers in the crystal. p202 HINb structure shows that two molecules form a face-to-face dimer via the same interface analogous to the HINa dsDNA binding site, and two such dimers further oligomerize tail to tail (Li et al., 2014a). The full-length p202 is also a tetramer *in vivo* and in *vitro*. It seems that the tetrameric HINb serves as the central platform for HINa to append to and increase the affinity of HINa for targeting dsDNA (Yin et al., 2013).

Additionally, due to p202 HINb interacting with AIM HIN, Yin et al. (2013) proposed that this interaction may result in a spatial separation of the AIM2 PYD and lead to p202 preventing ASC and AIM2 inflammasome activation. Li et al. (2014a) proposed a model of how the full-length p202 protein binds dsDNA from the crystal packing of the p202 HINa: dsDNA complex. Four p202 HINb domains form a tetramer and then tether four p202 HINa domains close, resulting in the simultaneous binding of p202 HINa domains to a dsDNA molecule.

### Molecular Mechanism of p202 Inhibiting Absent in Melanoma 2 Activation

To date, two molecular mechanisms of p202 negatively regulating AIM2 inflammasome have been proposed. In the model proposed by Yin et al. (2013), AIM2 HIN domains are spatially separated by the direct interaction between AIM2 HIN and p202 HINb, which prevents AIM2-mediated ASC oligomerization. The other mechanism was proposed by Ru et al. (2013) and Li et al. (2014a), they proposed that p202 inhibits the activation of AIM2 inflammasome by competition for dsDNA. Li et al. (2014a) found that the dsDNA affinity of p202 HINa is approximately fivefold higher than that of AIM2 HIN. The tetrameric p202 also enhances the binding affinity of the HINa domain for dsDNA. When p202 competes for dsDNA bound by AIM2, p202 HINa (higher DNA affinity) can displace AIM2 HIN from dsDNA. Subsequently, the free AIM2 HIN is recruited to and interacts with the closely linked p202 HINb tetramer, which would prevent AIM2 HIN from binding dsDNA and activating the AIM2 inflammasome. Combined with current research, both direct interactions between p202 HINb and AIM2 HIN and the competition of p202 HINa for DNA binding may play a role in p202 inhibiting the activation of AIM2 inflammasome (Li et al., 2014a).

### p204

Interferon-inducible protein 204 (p204) with an apparent molecular weight of 72 kDa is a mouse PYHIN protein and is a functional ortholog of human IFI16. p204 possesses PYD, HINa, and HINb domains (Figure 1). It modulates cell proliferation and differentiation (Luan et al., 2008; Zhao et al., 2015). It can directly interact with UBF1 to inhibit ribosomal RNA synthesis and interact with the Rb to coactivate certain transcription factors (Liu et al., 1999; Luan et al., 2007). Yi et al. (2018) found that p204 is necessary for lipopolysaccharide (LPS)-induced Toll-like receptor 4 (TLR4) signaling in pathogen infection. In recent years, p204 has been reported to act as a DNA sensor to defense against pathogen infections. It can sense viral DNA to activate the inflammasome and induce interferon production upon recognizing viral DNA in the cytoplasm and nucleus (Unterholzner et al., 2010; Conrady et al., 2012). It produces IFN-Is in cooperation with cGAS during bacterial infection (Storek et al., 2015). Knockdown of p204 can significantly inhibit IFN-β release in response to infections by *Francisella novicida* (Storek et al., 2015) and *Mycobacterium bovis* (Chunfa et al., 2017). Chen et al. (2019) reported that p204 triggers inflammatory responses during *Staphylococcus* infection. Ryabchenko et al. (2021) found p204 senses viral genomes in the nucleus during the infection of mouse polyomavirus (MPyV).

### Structural Basis of p204 Sensing Viral Double-Strand DNA

To date, crystal structures of p204 HINa, HINb, and HINab: dsDNA complex were reported (Tian and Yin, 2019; Fan et al., 2021). The overall structures of p204 HINa (PDB: 5YZP) and HINb (5YZW) are highly similar to those of known HIN domains (Figure 2B). The crystal structure of p204 HINab: dsDNA (PDB: 5Z7D) is the first complex structure of tandem HIN domain bound with dsDNA in the PYHIN family (Figure 2C). There are no obvious conformational changes of p204 HINa and HINb with or without dsDNA. p204 HINa and HINb share a similar dsDNA-binding mode in which the HIN domain binds to DNA mainly via the linker connecting two OB folds. This binding mode is the canonical dsDNA-binding mode of HIN domains in the PYHIN family (Figure 2D). The DNA-binding affinities of p204 HINa and HINb are similar. Furthermore, p204 HINab has a higher DNA-binding affinity than HINa or HINb alone, implying that HINa and HINb synergistically bind to dsDNA (Fan et al., 2021). Size exclusion chromatography and SAXS assay confirmed that p204 HINab alone stays as a monomer in the solution, similar to IFI16. In the p204 HINab: dsDNA complex structure, three HINab molecules form a C-ring-shaped structure for binding dsDNA. The linker between HINa and HINb changes ∼90° angles induced by dsDNA binding, resulting in higher dsDNA affinity (Fan et al., 2021). Interestingly, p204 HINa presents a dimer in the crystal structure, which attributes to dsDNA binding (Fan et al., 2021).

### Signal Transduction Pathway of p204

Combining several studies of the ortholog IFI16, the model of p204 recognizes viral dsDNA to activate downstream signaling pathways was proposed (Fan et al., 2021; Figure 3). p204 exhibits an extended conformation in the cytoplasm in the absence of dsDNA. In the presence of viral dsDNA, p204 HINa, and HINb would synergistically bind to dsDNA. Whereafter, more HINab molecules bind to dsDNA and form a C-ring-shaped structure along dsDNA. The dimerization of the HINa domain was stabilized by the binding of dsDNA, which results in the proximity of the adjacent N-terminal PYD domain. As an ortholog of human IFI16, p204 PYD may also aggregate and activate TBK1 and IRF3, inducing the production of IFNβ and proinflammatory cytokines to defend against viruses. The downstream signal pathway of p204 after recognizing viral DNA in the nucleus awaits further studies.

## Other Members of the PYHIN Family

### Myeloid Cell Nuclear Differentiation Antigen

Myeloid cell nuclear differentiation antigen (MNDA) is a 55 kDa protein and identified in HL-60 cells (Goldberger et al., 1986). It possesses an N-terminal PYD and a C-terminal HINa domain (Figure 1). It is expressed specifically in monocytes, myeloid progenitors, and granulocytes (Goldberger et al., 1984, 1986; Cousar and Briggs, 1990; Briggs et al., 1994). It can dimerize via an imperfect leucine zipper and a basic region (Xie et al., 1997a). Early studies reported that MNDA plays a role in myeloid differentiation and gene transcription. It can bind to the nucleolar proteins nucleolin and nucleophosmin (Xie et al., 1995, 1997b). It can also bind to dsDNA, and the interaction between MNDA and transcription factor YY1 can enhance the affinity of YY1 for its target DNA (Xie et al., 1998; Li et al., 2014b). MNDA plays a role in neutrophil apoptosis (Fotouhi-Ardakani et al., 2010). Latency-associated nuclear antigen (LANA) from KSHV has been shown to associate with MNDA, which may modulate IFN-mediated host defense activities (Fukushi et al., 2003). pUL83 can interact with the PYD of MNDA to block antiviral response (Li et al., 2013). Two crystal structures of MNDA PYD (PDB: 5WPZ and 5H7Q) have been solved, and these structures are highly similar to that of AIM2 PYD (Figure 2A).

### Interferon-Inducible Protein X

Interferon-inducible protein X (IFIX, also know as PYHIN1) gene is predicted to encode six protein isoforms (α1, α2, β1, β2, γ1, and γ2) which are localized in the nucleus (Ding et al., 2004). All of them have a PYD and an NLS. Except for IFIXγ, both IFIXα and IFIXβ have HINa domain (Figure 1). Ding et al. (2004) found that IFIXα1 possesses antitumor activity and proposed that it be used as a therapeutic agent. They also found that IFIXα1 can interact with HDM2 to positively regulate p53, which may contribute in part to the antitumor activity (Ding et al., 2006). Li et al. (2013) found that pUL83 can interact with IFIX PYD to block its antiviral response. Diner et al. (2015) found that IFIX can restrict herpesvirus replication via binding viral DNA with the HIN domain and leads to IFN-I response. They also discovered that IFIX could act as an antiviral DNA sensor to detect viral DNA in the nucleus and cytoplasm (Diner et al., 2015). The molecular mechanism of dsDNA recognition and activation for IFIX awaits future studies.

### Pyrin Domain Only Protein 3

Pyrin domain only protein 3 (POP3) is sometimes considered a new member of the PYHIN family and was identified in 2014 (Connolly and Bowie, 2014; Khare et al., 2014). It only possesses a PYD and lacks the typical HIN domain of the PYHIN family (Figure 1). It could interact with the PYDs of AIM2 and IFI16, resulting in the inhibition of DNA virus-induced activation of AIM2 and IFI16-inflammasomes (Khare et al., 2014). However, the molecular mechanism of POP3’s interaction with the PYDs of AIM2 and IFI16 remains unknown.

### p203

p203 is a nuclear protein in mice and was identified in 1997 (Gribaudo et al., 1997). It possesses PYD and HINb domains (Figure 1). It is expressed in the thymus, bone marrow, spleen, and liver (Gribaudo et al., 1999; Zhang et al., 2008). The level of p203 protein decreased during liver regeneration (Zhang et al., 2008).

### p205

p205 harbors a PYD and a C-terminal HINa domain (Figure 1). It is expressed in the thymus, bone marrow, spleen, heart, and skeletal muscle tissue (Asefa et al., 2004; Weiler et al., 1999). It contributes to cell growth and allows progenitor cells to differentiate during myelomonocytic cell differentiation (Dermott et al., 2004). It can directly bind to Rb and p53 and upregulate p21 and Rb (Asefa et al., 2006). It can also inhibit growth in proliferating cells (Asefa et al., 2006). The solution structure of p205 PYD has been solved (PDB: 2YU0) (Figure 2A), which is highly similar to known PYD.

## Comparison of HIN Domains in PYHIN Family

To date, the known HIN domain structures include human AIM2 HIN (Jin et al., 2012), human IFI16 HINa and HINb (Liao et al., 2011; Jin et al., 2012; Ni et al., 2016), mouse AIM2 HIN (Ru et al., 2013), mouse p202 HINa and HINb (Ru et al., 2013; Yin et al., 2013), and mouse p204 HINa and HINb (Tian and Yin, 2019; Fan et al., 2021). Although the overall structure of HIN domains is highly conserved, their superposition reveals significant flexibility in the loops in OB folds (Figure 2B). Furthermore, these HIN domains exhibit different surface charges, indicating distinct DNA-binding surfaces (Fan et al., 2021).

Six HIN: dsDNA complex structures have been reported, including human AIM2: dsDNA (PDB: 3RN2) (Jin et al., 2012), mouse AIM2: dsDNA (PDB: 4JBM) (Ru et al., 2013), human IFI16 HINa: dsDNA (PDB: 4QGU) (Ni et al., 2016), human IFI16 HINb: dsDNA (PDB: 3RNU) (Jin et al., 2012), mouse p202 HINa: dsDNA (Ru et al., 2013; Yin et al., 2013), and p204 HINab: dsDNA (PDB: 5Z7D) (Fan et al., 2021). The structural superposition of the HIN: dsDNA complex reveals two distinctly different DNA-binding modes in the PYHIN family (Figures 2D,E). AIM2 HIN, IFI16 HINb, p204 HINa, and HINb employ the linker connecting two OB folds and the surrounding residues to engage dsDNA, whereas IFI16 HINa and p202 HINa interact with dsDNA via an opposite surface formed by the loops of two OB folds.

## Therapeutic Approaches to Targeting PYHIN Proteins

Concerning their roles in innate immune responses, PYHIN family members could be utilized as therapeutic targets in autoimmune, inflammatory disorders, and cancers. For example, synthetic oligodeoxynucleotides (ODN) A151 can inhibit immune responses by binding to AIM2, which may treat infectious and autoimmune diseases (Kaminski et al., 2013). LL-37, a human cathelicidin antimicrobial peptide, can interact with DNA to block AIM2 inflammasome activation, which could be used to treat chronic skin disease (Dombrowski et al., 2011). A peptide derived from a virus may aid the design of inhibitors. Such as A46 (known as VIPER), a peptide derived from VACV, could inhibit the TLR signaling pathway (Lysakova-Devine et al., 2010). Thus, analogical viral inhibitors could be designed to target PYHIN proteins. Cai et al. (2020) found that IFI16 can upregulate PD-L1 through the STING-TBK1-NF−κB pathway to promote cervical cancer progression, suggesting IFI16 could be developed as a novel immunotherapy target.

## Concluding Remarks and Perspective

Immunological studies, in association with biochemical and structural studies, have revealed the molecular mechanisms of AIM2, IFI16, p202, and p204, sensing viral DNA and resulting in immune responses. However, several questions remain unclear. Firstly, what is the molecular basis for DNA length-dependent response? Morrone et al. (2014) suggested that even in the presence of excess DNA, the formation of IFI16 PYD-driven filament could allow multiple IFI16 to bind adjacent molecules to form signaling foci. Secondly, why IFI16 does not bind to self-DNA? Morrone et al. (2014) suggested that due to the weak binding affinity of HIN domains to self-DNA, HIN domains coupled with filament formation could sufficiently inhibit the interaction with self-DNA. In addition, they also suggested that although the viral genome and host histones package into chromatin after virus invasion, it is more loosely packed than self-DNA. Furthermore, IFI16 filament selectively engages foreign DNA while minimizing its interaction with self-DNA by a competitive mechanism. It is still needed to investigate these fundamental questions further. Thirdly, the mechanism of AIM2 recognizing viral RNA has yet to be investigated. PYHIN proteins participate in response to many pathogens and mediate their clearance. They also contribute to the pathogenesis of autoimmune, auto-inflammatory diseases and cancers. Investigation into the therapeutic approaches targeting PYHIN proteins will greatly aid the development of treating associated diseases.

## Author Contributions

XF wrote the manuscript. LJ and TJ reviewed and edited the manuscript. All authors provided funding and contributed to the article, and approved the submitted version.

## Conflict of Interest

The authors declare that the research was conducted in the absence of any commercial or financial relationships that could be construed as a potential conflict of interest.

## Publisher’s Note

All claims expressed in this article are solely those of the authors and do not necessarily represent those of their affiliated organizations, or those of the publisher, the editors and the reviewers. Any product that may be evaluated in this article, or claim that may be made by its manufacturer, is not guaranteed or endorsed by the publisher.
